# 

**Published:** 1894-01-27

**Authors:** 


CONTENTS.

**Imperial Typhoid: The Unemployed** - 275
**Around the Hospitals** - 276
**On Modern Progress in Ophthalmic Medicine and Surgery.** By ROBERT BRUDENELL CARTER - 277
**Art and Disease.** III.—Modified Forms of Possession - 278
**Modern Medico-Psychology and Psychiatry.** By T. S. CLOUSTON, M.D. I.—Introductory - 279
**The History and Capabilities of Herbal Simples,** By W. T. FERNIE. Goose-Grass - 280
**Annotations.**—Private: for Doctors only—Undignified Preventive Medicine—Materialism, Madness, and Poor Humanity - 281
**Notes and News** - 282
**Editor's Letter-Box.**—Midwives Registration Association—A Munificent Gift - 294
**Medical Progress and Hospital Clinics**—
LONDON HOSPITAL.—The Drainage of Wounds and Cavities - 283
Thyroid Feeding in Skin Diseases - 284
Diphtheria: Some Points in the Pathology and Treatment - 285
MEDICAL PROGRESS—Hæmoptysis - 287
Disease of the Vermiform Appendix - 288
NEW DRUGS AND PREPARATIONS.—Chlaralose - 290
**The Institutional Workshop**—
HOSPITAL FINANCE. II.—Endowments - 291
THE STORY OF THE INSANE FROM YEAR TO YEAR - 292
PRACTICAL DEPARTMENTS—Appliances at Poplar (*continued*) - 293
THE HOSPITAL SATURDAY FUND - 293
**Scraps and Gleanings** - 294
**The Book World of Medicine and Science** - 2.5

EXTRA SUPPLEMENT.—THE NURSING MIRROR.

**News from the Nursing World.**—Westminster Hospital Samaritan Fund—After-Care Association—Helps Homewards—Repeated Warnings—A Day of Reckoning—An Overflow—Better Late than Never—Queen's Nurses—Nurses at the Rotunda—A Scotch Co-operation—Proposed Progress at Tiverton—Hospital for Sick Children—Short Items - clxi
**On Nursing the Nervous and Insane.** III.—Uses of Electricity in Nervous Diseases - clxiii
**Appointments—Minor Appointments** - clxiii
**District Nursing.**—IV. District Hygine - clxiv
**Stray Visitors—Death in our Ranks—Where to Go** - clxv
**Nursing in Argentina.**—School for Male and Female Nurses - clxvi
**Women's Work.**—As Medical Students - clxvii
**The Real and Ideal Nurse** - clxvii
**Novelties for Nurses** - clxviii
**The German Hospital, Dalston** - clxviii
**Primitive Massage—Notes and Queries** - clxviii
**The Muses' Looking Glass.**—The "Old Masters" at Burlington House—The Exhibition of Early Italian Art - clxix

